# Function of γδ T cells in tumor immunology and their application to cancer therapy

**DOI:** 10.1038/s12276-021-00576-0

**Published:** 2021-03-12

**Authors:** Jang Hyun Park, Heung Kyu Lee

**Affiliations:** Graduate School of Medical Science and Engineering, Korea Advanced Institute of Science and Technology (KAIST), Daejeon, 34141 Republic of Korea

**Keywords:** Innate immune cells, Tumour immunology

## Abstract

T cells of the γδ lineage are unconventional T cells with functions not restricted to MHC-mediated antigen presentation. Because of their broad antigen specificity and NK-like cytotoxicity, γδ T-cell importance in tumor immunology has been emphasized. However, some γδ T-cell subsets, especially those expressing IL-17, are immunosuppressive or tumor-promoting cells. Their cytokine profile and cytotoxicity are seemingly determined by cross-talk with microenvironment components, not by the γδTCR chain. Furthermore, much about the TCR antigen of γδ T cells remains unknown compared with the extreme diversity of their TCR chain pairs. Thus, the investigation and application of γδ T cells have been relatively difficult. Nevertheless, γδ T cells remain attractive targets for antitumor therapy because of their independence from MHC molecules. Because tumor cells have the ability to evade the immune system through MHC shedding, heterogeneous antigens, and low antigen spreading, MHC-independent γδ T cells represent good alternative targets for immunotherapy. Therefore, many approaches to using γδ T cells for antitumor therapy have been attempted, including induction of endogenous γδ T cell activation, adoptive transfer of expanded cells ex vivo, and utilization of chimeric antigen receptor (CAR)-T cells. Here, we discuss the function of γδ T cells in tumor immunology and their application to cancer therapy.

## Cancer: T cell subset could boost immunotherapy

An under-researched subset of T cells may find application in cancer immunotherapy once their multiple roles are fully understood. Jang Hyun Park and Heung Kyu Lee at the Korea Advanced Institute of Science and Technology, Daejeon, South Korea, have reviewed the current understanding of gamma delta (γδ) T cells, which are found in peripheral blood and specific tissues, for example, the skin. They form part of the immune response, but instead of recognizing antigens presented by MHC molecule as do other T cells, they respond to markers of cellular stress during events such as tumorigenesis. Evidence suggests γδ T cells play different roles in the tumor microenvironment depending on the proteins they produce and can induce anti-tumor or pro-tumor activity. The authors suggest that harnessing the anti-tumor properties of γδ T cells for use in immunotherapy could transform cancer treatments.

## Introduction

The novel T-cell γδ line was identified upon the discovery of the γ gene in 1984^1,2^. Furthermore, γδ T cells express γδ T-cell receptor (γδTCR) but not αβTCR. γδ T cells constitute part of the “unconventional” T-cell subset and function in unique roles, such as stress surveillance^3^. The most unique characteristics of γδ T cells are migration to peripheral tissues rather than lymphoid organs and functions independently of major histocompatibility complex (MHC)-dependent antigen presentation^4^. In mice, γδ T cells first develop in the embryonic thymus. Compared with conventional T cells, which are derived from double-positive (DP) thymocytes, γδ T cells are derived from CD4^−^/CD8^−^ double-negative (DN) thymocytes. From the DN stage, functionally distinct γδ T cells develop at different stages along with varying TCR pairs^5^. The anatomical localization of γδ T cells is also different from each other. For example, Vγ5^+^ dendritic epidermal T cells (DETCs; Tonegawa nomenclature) are located in the epidermis of the skin, whereas Vγ6^+^ cells reside in the meninges, genital tract, and lungs. Vγ4^+^ cells are located in the liver, lymphoid organs, and skin, whereas Vγ7^+^ intraepithelial lymphocytes (IELs) are located in the gut. Vγ1^+^/Vγ4^+^ cells are generated during and after the postnatal period and are distributed systemically, similar to adaptive immune cells^6^. Because γδ T-cell subsets are not conserved between mice and humans, the translation of results is difficult. For example, DETCs do not exist in human skin. Usually, γ chains are used to classify murine γδ T cell subsets; however, δ chains are used to classify these sets in humans^7^. Furthermore, γδ T cells constitute a minor population in both mice and humans. However, they participate in host defense against a variety of conditions, including viral and bacterial infections and cancer^8^. Specifically, due to their strong cytotoxicity and unrestricted MHC features, γδ T cells are thought to be a good alternative therapeutic target for cancer^7^. In this review, we discuss the complex potential role of γδ T cells in the tumor microenvironment (TME) and the possibility of using γδ T-cell-based antitumor immunotherapy in the future.

## The antitumor function of γδ T cells

### Direct cytotoxicity

**Fig. 1:**
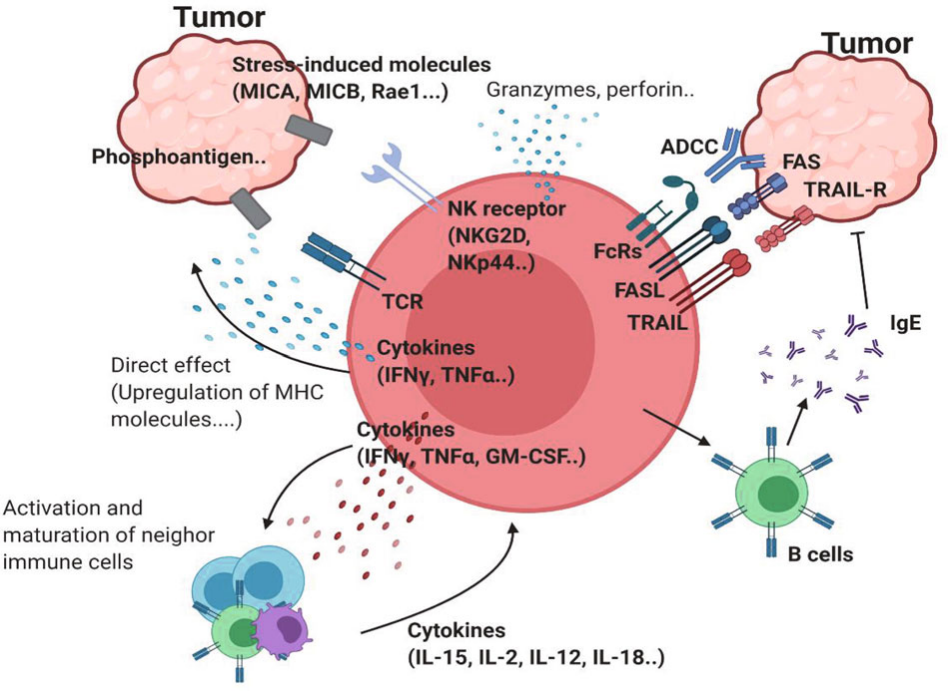
Network of antitumor γδ T cells. γδTCR ligands such as phosphoantigens can bind to γδTCR. Stress-induced molecules, including MICA/B and Rae-1, can bind to NK receptors such as NKG2D. This ligation induces activation of antitumor γδ T cells. Proinflammatory cytokines, such as IFNγ and TNF, can further activate antitumor immunity by inducing MHC molecules on the tumor cell surface or by affecting other immune cells. The upregulation of cytotoxic molecules such as granzymes and perforin can directly kill tumor cells. In addition to these receptors, FcR-mediated ADCC, FAS-FASL, and TRAIL ligation can also induce direct cytotoxicity against tumor cells. γδ T cells can promote B cells to produce IgE, which has an antitumor effect.

γδ T cells are frequently observed in multiple tumor tissues, and their presence is thought to be a favorable prognostic factor^9^. In addition, γδ T cells are known as stress sensors. Ligation between stress-induced molecules, such as MHC class I polypeptide-related sequence A (MICA) and natural killer group 2 member D (NKG2D), provokes target-specific killing^10^. Transformation is one cellular stress mechanism that induces the expression of NKG2D ligands (NKG2DLs)^11^. Thus, γδ T cells are generally considered cytotoxic and antitumor lymphocytes (Fig. 1). The Hayday group showed that γδ T cells can recognize and regulate cutaneous malignancy using PDV cell line injection and methylcholanthrene (MCA)- and dimethylbenz[a]anthracene (DMBA)-induced cutaneous tumors^12^. The antitumor function of γδ T cells has been extended to other tumors, such as B-cell lymphoma, prostate cancer, melanoma, and mesenchymal glioblastoma^13–16^. In addition to NKG2D, γδ T cells need various types of receptors depending on the context. For example, γδ T cells need both γδTCR and NKG2D to kill TCCSUP human transitional cell carcinoma cells^17^. However, γδ T cells need only γδTCR to target the zoledronate-treated human rhabdomyosarcoma (RMS) cell line^18^. Daudi cells, which express endogenous γδTCR ligands but not MHC class I or MICA ligands, are not dependent on NKG2D. Furthermore, the RMA murine lymphoma cell line does not express NKG2DL^19^. Other NK receptors (NKRs), including CD226 (DNAM-1), natural cytotoxicity-triggering receptor 3 (NCR3; NKp30), and NCR2 (NKp44), also participate in tumor recognition^20^. TNF receptors, such as TNF-related apoptosis-inducing ligand (TRAIL) and Fas ligand (FASL), can also kill tumor cells^7^. Human γδ T cells express CD16 and participate in inducing antibody-dependent cellular cytotoxicity^21^.

### Interaction between the microenvironment and γδ T cells

Certain environments regulate γδ T cells for optimal activation. For example, IL-2 and IL-15 are the main inducers of cytotoxic γδ T cells^22^. IL-12, IL-18, IL-21, and IL-36γ are also involved in IFNγ production and cytotoxicity^7^. In contrast, IL-10 and transforming growth factor-beta (TGFβ) secreted from regulatory T cells or myeloid cells can negatively regulate γδ T cells^23^. Although γδ T cells are known to function independent of MHC recognition, MHC-restricted γδTCRs in MART1- and gp100-reactive γδ T cells have been reported^24^. Environmental factors also affect γδ T-cell function. For example, reactive oxygen species, oxygen tension, and cholesterol can influence IFNγ and/or NKR expression^25–27^. In the case of high-grade glioma, tumor-infiltrated γδ T cells have a high apoptosis rate. The number of γδ T cells decreases dramatically at the terminal stage of the disease^28^. These results suggest that the TME attenuates γδ T-cell responses in multiple ways. Because γδ T cells are actively recruited to the TME and have the potential to kill target cells in vitro, our lack of understanding of how the TME suppresses γδ T cells remains a major challenge.

### γδ T cells bridge innate and adaptive antitumor responses

Because γδ T cells are early stress responders and possess adaptive immunity-like characteristics, they are thought to create a link between innate and adaptive immune responses. γδ T cells comprise various subsets categorized by their combination of TCR chains. SRY-box transcription factor (*Sox13*)^+^ fetal progenitors for Vγ2^+^ T cells originate independently of γδTCR^29^. Subsets of innate-like γδ T cells do not require TCR engagement but might be dependent on cytokines or innate receptors, such as NKG2D. However, γδ T cells usually need a TCR signal for their activation and development. Studies have demonstrated the effect of different antigens on γδ T-cell development and activation^30,31^. Furthermore, persistent expansion of γδ T cells in cytomegalovirus (CMV)-infected patients following kidney transplantation has been observed^32^. Proinflammatory αβ- and γδTCR-coexpressing T cells have also been reported^33^, suggesting that subsets of γδ T cells possess adaptive, not innate, features. Research using tuberculosis infection in nonhuman primates has shown that Vγ9Vδ2 T cells can induce memory responses^34^. Thus, γδ T cells have both innate and adaptive functions and can link both responses^6^. In cases of cancer, it is still unclear whether γδ T cells act more innately or adaptively. However, we hypothesize that innate-like γδ T cells may acutely respond to tumor cells via stress sensing, whereas adaptive γδ T cells may establish durable antitumor responses in an antigen-specific manner. Because γδ T cells have been shown to expand following CMV infection, CMV-positive tumor cells may be good models for investigating adaptive γδ T cells. CMV-specific T cells are reactive to glioblastoma multiforme (GBM) cells^35^. Thus, although controversial, cell therapy using γδ T cells against CMV-positive tumors may be applicable. On the other hand, γδ T cells are an early source of IFNγ in the TME^36^. IFNγ derived from γδ T cells can amplify the production of αβ T cells and induce the expression of MHC class I molecules on tumor cells^37^. In addition, antigen presentation and costimulation of αβ T cells derived from γδ T cells have been observed in gastric cancer^38^. The antigen-specific T-cell expansion has been successfully induced by coculture with γδ T cells^39^. In addition to CD4 T cells, γδ T cells can also boost B cells. Topical 12-dimethylbenz[a]anthracene (DMBA)-induced tumorigenesis promoted B-cell IgE production in a Vγ5^+^ T cell-dependent manner. IgE was also shown to protect a host from carcinogenesis^40^. GM-CSF produced by γδ T cells controlled CD103^+^ dendritic cells (DCs)^41^. These findings show that γδ T cells can communicate with multiple immune cells surrounding the TME. In summary, although γδ T cells are considered innate immune cells, subsets of γδ T cells exhibit adaptive characteristics and can serve as a bridge between innate and adaptive immune responses.

## The protumor function of γδ T cells

### The protumor function of IL-17-producing γδ T cells

**Fig. 2:**
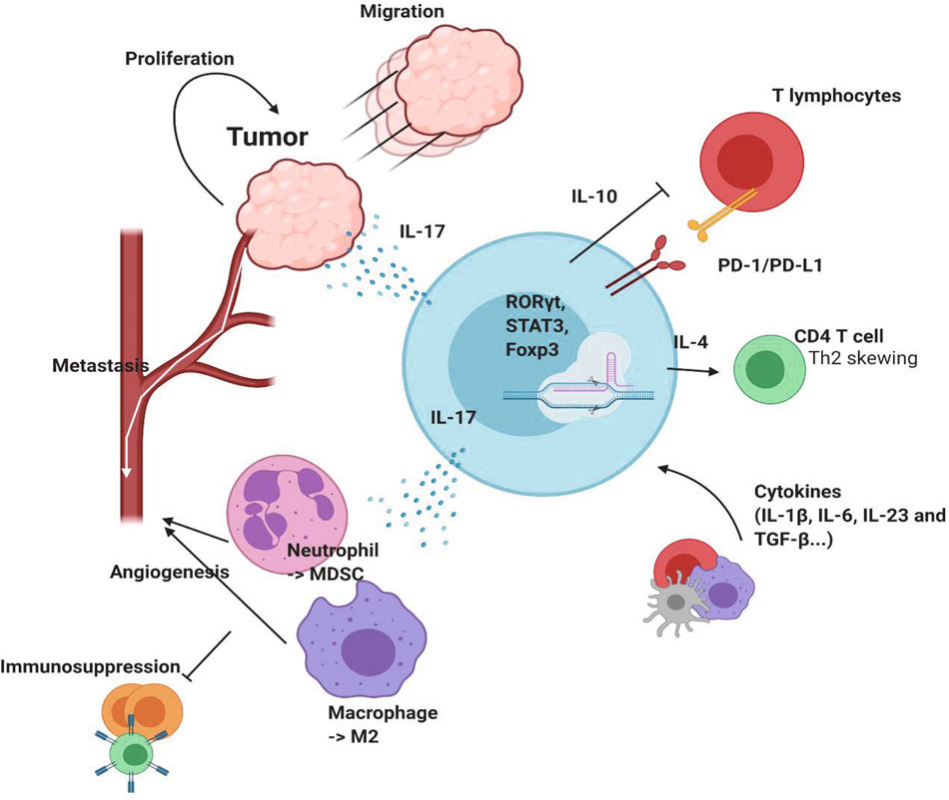
The role of protumor γδ T cells. Tumor-promoting γδ T cells express RORγt and STAT3 to promote IL-17 production, as well as Foxp3, which is a marker of regulatory T cells. IL-17 secreted from γδ T cells promotes tumor cell proliferation and migration, which can provoke metastasis to distant organs. γδ T cell-derived IL-17 induces MDSC differentiation from granulocytes such as neutrophils and the M2 phenotype acquisition of macrophages. γδ T cells promote metastasis through angiogenesis and suppress immune cells. IL-4 produced from tumor-promoting γδ T cells skews CD4 T cells to differentiate into Th2 cells and inhibits Th1 responses. IL-10 secreted from γδ T cells further inhibits T cells. In addition, surface expression of PD-L1 attenuates PD-1-expressing lymphocytes.

In general, IL-17A-producing γδ T cells are considered to be tumor-promoting cells (Fig. 2). IL-17-producing γδ T cells are rarely found in healthy humans^42^. However, in multiple tumor models, tumor injection induces IL-17A production by γδ T cells. Furthermore, IL-17-deficient animals show reduced tumor mass in breast cancer, hepatocellular carcinoma, lung cancer, and melanoma^7^. The tumor-promoting function of IL-17A is mainly manifested by angiogenesis and metastasis. In fibrosarcoma, circulating γδ T cells, but not Vγ5^+^ cells, produce IL-17A and promote angiogenesis^43^. In mice, IL-17 is usually produced by Vγ4^+^ or Vγ6^+^ cells^44^. In humans, although IL-17 can be secreted by Vγ9Vδ2^+^ T cells upon stimulation with antigens and cytokines, such as IL-1β, IL-6, IL-23, and TGFβ^45^, IL-17 has been shown to be preferentially produced by Vδ1^+^ T cells^46^. Because tissue-resident innate-like γδ T cells are more prone to producing IL-17 than circulating γδ T cells, which preferentially produce IFNγ, tissue-resident Vδ1^+^ T cells, not Vδ2^+^ T cells, may be main sources of IL-17. However, biology seems is complicated. The cytokine profile of γδ T cell subsets is highly dependent on context. In breast cancer, tissue-resident Vδ1^+^ T cells are skewed toward cytolysis and IFNγ production but not IL-17 production^47^. Thus, the determination of whether γδ T cells produce IL-17 or IFNγ based on TCR chains might be meaningless. IL-17 contributes to tumor progression in multiple ways. As mentioned above, IL-17 can promote angiogenesis through direct signaling on endothelial cells^43^. However, IL-17 can promote angiogenesis indirectly. IL-17 can promote macrophages to make angiogenic factors such as vascular endothelial growth factor^48^. Furthermore, IL-17 can induce M2 macrophage polarization^49^. In addition, γδ T cells can recruit neutrophils and facilitate their expansion in the TME through IL-17 and G-CSF^50^. On the other hand, through the PI3K/AKT signaling pathway, IL-17 can directly activate tumor cells to be more migratory^51^. Despite these findings, the roles of IL-17 remain unclear, depending on the tumor model and specimen. For example, two studies focusing on colon cancer have shown opposite conclusions. One study suggested that high numbers of γδ T cells reside in the TME and act as the main sources of IL-17^52^. However, the results from another study suggest that γδ T cells are major sources of IFNγ, not IL-17, and constitute a very minor population^53^. Studies focusing on brain tumors are present contradictory findings. IL-17A can promote the migration of U87 MG and U251 human GBM cells^51^, and inhibition of IL-17A can extend the overall survival of patient-derived tumor-bearing immunodeficient mice^54^. However, one study showed that GBM patients who express high levels of IL-17 survive longer than those expressing lower levels^55^. These data suggest that the role of IL-17 varies depending on the context and network of cells in the TME.

### Other mechanisms of tumor-promoting γδ T cells

In addition to IL-17, other mediators secreted from γδ T cells can promote tumor progression. CD39^+^ γδ T cells can suppress immune responses through the adenosine pathway and recruit myeloid-derived suppressor cells in colorectal cancer^56^. These CD39^+^ γδ T cells express FOXP3, a marker of regulatory T cells. TGFβ treatment increases FOXP3 expression in human peripheral blood mononuclear cell (PBMC)-derived γδ T cells. FOXP3^+^ γδ T cells can inhibit the proliferation of T cells derived from PBMCs^57^. In a murine sarcoma model, γδ T cells secrete galectin-1, which suppresses cytotoxic CD8 T cells^58^. In murine pancreatic cancer, γδ T cells express programmed death-ligand 1 (PD-L1) and galectin-9 to suppress cytotoxic T cells^59^. IL-4-conditioned γδ T cells are more likely to form a subset of Vδ1^+^ T cells that inhibit T-cell proliferation in an IL-10-dependent manner^60^.

## Regulation of γδ T cells

### Recruitment of γδ T cells

CC-chemokine receptor 6 (CCR6) is a well-defined chemokine receptor of IL-17-producing γδ T cells. Vγ4^+^ and Vγ6^+^ T-cell localization to the skin is dependent on CCR6. However, activation of IL-17-producing γδ T cells leads to the downregulation of CCR6 in an IL-1β-, IL-23-, and/or IL-7-manner. Because IL-17-producing γδ T cells coexpress CCR2, CCR6 downregulation promotes CCR2 dependency. The migration of γδ T cells into inflammatory sites has been shown to be dependent on CCR2^61^. Furthermore, IFNγ-producing γδ T cells also express CCR2. However, blood-derived Vδ1^+^ T cells, but not Vδ2^+^ T cells, express CCR2^62^. Vδ1^+^ T cells also express CXCR3; however, Vδ2^+^ T cells distinctly express CCR5^63,64^. In the TME, γδ T cells seem to be recruited mainly by CCR2 and CXCR3^65^. However, an accurate characterization of chemokine receptor expression on γδ T cells remains to be performed. Adhesion molecules, including LFA-1, are also important for γδ T-cell recruitment^66^.

### T-cell receptor signaling

**Fig. 3:**
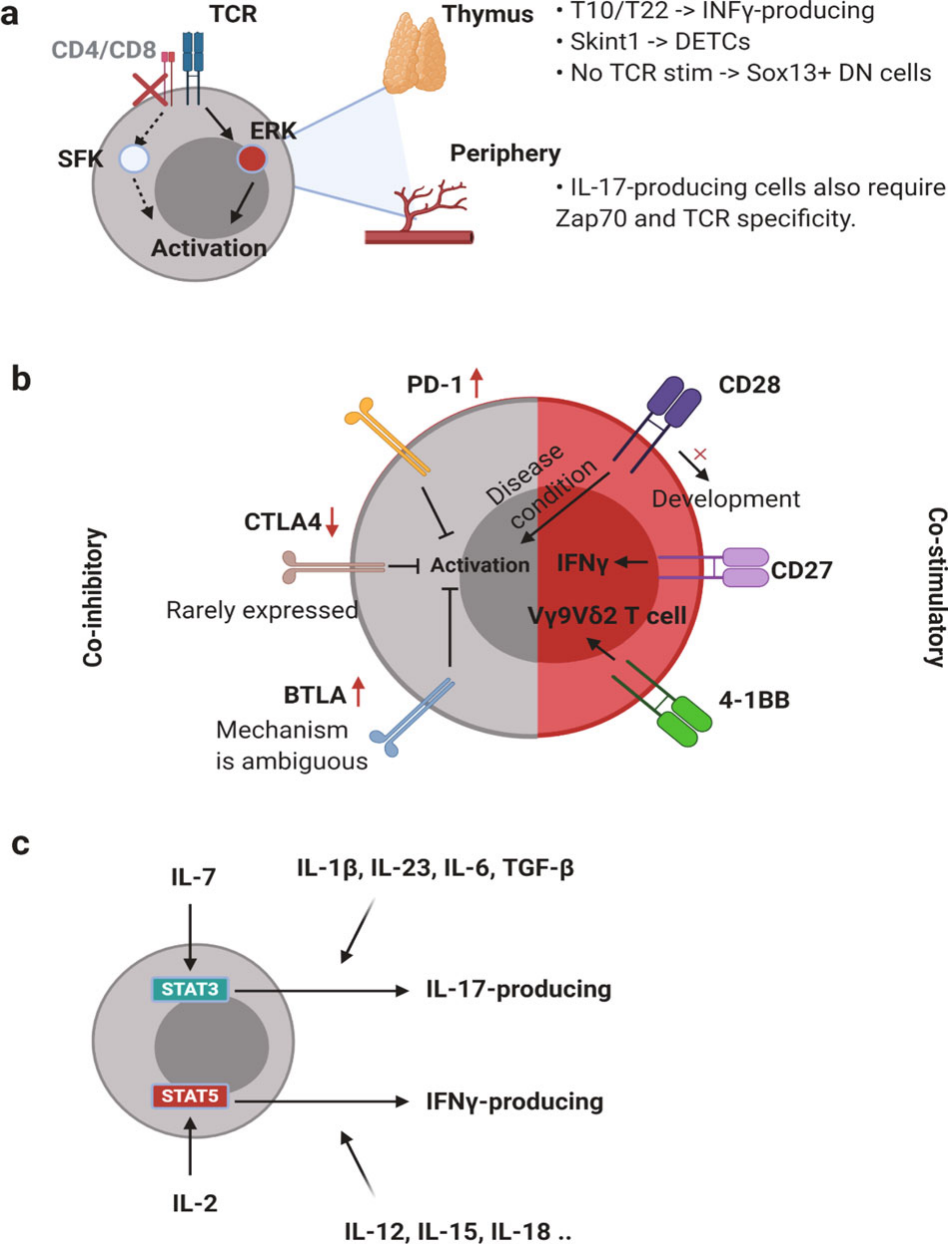
Regulation of γδ T cells. **a** Although there are exceptions, γδ T cells generally do not express CD4 or CD8. Therefore, SFK-mediated activation cannot be used to activate γδ T cells. However, the ERK pathway is activated. For the normal development of γδ T cells, TCR signaling is important. T10/T22 and Skint1 are well-defined antigens for γδTCR. T10/T22-mediated signaling induces IFNγ-producing γδ T cells, whereas the Skint1 signaling induces DETC development. Some innate subsets of γδ T cells do not require TCR stimulation during development. Although several reports have demonstrated that innate IL-17-producing γδ T cells do not require TCR signaling, other studies have shown that TCR specificity or Zap70 phosphorylation is needed for full γδ T cell activation. **b** Coinhibitory molecules, including PD-1, CTLA4, and BTLA, can suppress γδ T cells. PD-1 and BTLA are frequently expressed by γδ T cells. However, CTLA4 is rarely expressed on the γδ T cell surface. Costimulatory molecules such as CD28, CD27, and 4-1BB are needed for development or activation. CD28 is needed for advancing disease conditions but not its development. However, CD27 is needed for the development and proper expression of IFN-γ. 4-1BB is known to enhance Vγ9Vδ2 T cells. However, the roles and mechanisms of these and other coinhibitory/stimulatory molecules remain unclear. **c** Various cytokines induce γδ T cell differentiation, leading to different functions. The γδ T-cell differentiation dichotomy is mainly affected by cytokine signals.

The γδTCR complex is composed of γδTCR and other CD3 chains. TCR signal strength is important for γδ T-cell development and activation (Fig. 3a). A strong TCR signal through γδTCR causes αβ/γδ common precursors to differentiate into γδ T cells^67^. In general, ligated TCR complexes induce the phosphorylation of immunoreceptor tyrosine-based activation motifs (ITAMs) by SRC family kinases (SFKs), lymphocyte-specific protein tyrosine kinases (LCKs), and FYN proto-oncogenes (FYNs). However, although in contrast to other T cells, γδ T cells do not express CD4 or CD8; therefore, the mechanism by which TCR signaling is mediated remains unclear. One possible explanation involves extracellular signal-regulated kinase (ERK) phosphorylation, which can activate SFKs^68^. The requirement for TCR signaling is dependent on context (cytokines, inflammation, etc.) or cellular subsets defined by their γ and δ chains. IFNγ-producing γδ T cells, but not IL-17-producing γδ T cells, require the thymic expression of T10/T22^30^. Thymic selection and maintenance of intraepithelial T-cell protein 1 (Skint1) also lead to IFNγ-producing DETCs, not to IL-17 production, because it suppresses SOX13 and RORγt^31^. *Sox13*-expressing DN1d thymocytes, which are progenitors of IL-17-producing γδ T cells, do not require TCR expression or signaling. However, phycoerythrin (PE)-specific γδ T cells were shown to secrete IL-17 in a TCR-dependent manner. In addition, signaling through the zeta chain of T cell receptor-associated protein 70 (ZAP70) is required for the development of IL-17-producing γδ T cells but not IFNγ-producing γδ T cells^69^. In summary, the requirement for TCR signaling is dependent on subsets, context, and antigens.

Recent interesting research has suggested that abrogating γδTCR rearrangements lead to innate lymphoid cell 2 (ILC2) generation. TCRδ-deficient mice showed an increase in ILC2s. Thus, we need to be careful when interpreting phenotypes using TCRδ-deficient mice^70^. Because the identity of general antigens for γδ T cells remains unclear, knowledge about specific tumor antigens is even nebulous. Annexin A2, which can bind to the Vδ3 TCR, is expressed in multiple cancer cell types, including endometrial, breast, and glioblastoma cells^71^. p-Ag-bound butyrophilin 3A (BTN3A) isoforms that can activate Vγ9Vδ2 T cells through TCR signaling to play critical roles in regulating antitumor immunity^72^. Various kinds of ligands of γδTCR (Annexin A2, tRNA synthetases, T10/T22, Skint-1, etc.) are currently being discovered, and they are expressed by multiple cancer cell types^73^. The identification of antigens is difficult because of the relatively low affinity of TCRs. If we can identify and categorize ligands similar as we did with vitamin B-MAIT cells, lipid antigen-NKT cells, and peptide antigen-αβ T cells, our understanding of γδ T-cell involvement in antitumor immunity will be greatly enhanced.

### Costimulatory and inhibitory molecules

Costimulatory molecules prevent anergy and enhance T-cell activation (Fig. 3b). CD28, the most well-understood costimulatory molecule, is expressed on γδ T cells and promotes proliferation and survival through IL-2 signaling^74^. However, the requirement for CD28 signaling differs depending on the disease stage and/or tumor type^74,75^. Thus, the requirement should be determined accordingly. CD28 is not necessary for the development of γδ T cells. However, CD27 is important for IFNγ-producing γδ T cells^76^. CD27 is often used as a marker for IFNγ-producing γδ T cells because CD27-dependent division of γδ T cells occurs in the thymus. However, its role in the TME remains unclear. Because the CD70-CD27 interaction can enhance IFNγ production and the survival of Vγ9Vδ2 T cells in vitro^77^, it might be an applicable target in antitumor γδ T-cell therapy. CD137 (4-1BB) can enhance Vγ9Vδ2 T-cell function following influenza or *Listeria* infection. Thus, CD137 may also be involved in the antitumor function of γδ T cells^78,79^.

The reinvigoration of exhausted or inhibited T cells is an increasingly studied approach to immunotherapy. Because γδ T cells play crucial roles in various types of tumors, understanding the inhibitory signal of γδ T cells might be beneficial for developing effective immunotherapies. Although γδ T cells rarely express cytotoxic T-lymphocyte-associated protein-4 (CTLA-4), upon activation, γδ T cells rapidly upregulate programmed cell death protein-1 (PD-1) and B- and T-lymphocyte attenuator (BTLA)^80^. Although PD-1 signaling exerts an inhibitory effect on IFNγ production, phospho-antigen-mediated activation of Vγ9Vδ2 T cells can overcome PD-1 signaling^81^. Other costimulatory molecules, such as OX40 and CD40L, and coinhibitory molecules, including V-domain immunoglobulin suppressor of T cell activation (VISTA), T-cell immunoreceptor with Ig and ITIM domains, or glucocorticoid-induced TNFR-related protein, might be involved in γδ T-cell activation in various ways. Thus, a comprehensive investigation of these molecules is needed to develop effective γδ T cell-based therapies.

### Cytokines

Cytokines such as IL-2 and IL-7 are essential for the survival and proliferation of T cells (Fig. 3c). Additional cytokines, including IL-4 and IL-12, are important for determining the differentiation fate of T cells. IL-7 and IL-15 have been established as the most important cytokines for murine γδ T-cell development and homeostasis. Conditional depletion of IL-7 from thymic epithelial cells resulted in a reduction in γδ T cells in multiple organs, such as the thymus, gut, and skin^82^. IL-7 is seemingly important for the early development of γδ T cells, especially IL-17-producing γδ T cells. IL-7 facilitates the selective expansion of IL-17-producing γδ T cells through signal transducer and transcription 3 (STAT3)-dependent signaling. This signaling pathway induces γδ T-cell resistance to activation-induced cell death and proliferation^83^. However, gut-residing intraepithelial γδ T cells tend to be relatively more dependent on IL-15^84^. On the other hand, IFNγ-producing γδ T cells require IL-15 and IL-2, but not IL-7^22^. In addition to phospho-antigen-dependent TCR activation, IL-2 and IL-15 activate γδ T-cell differentiation even though TCR signaling is dispensable for ERK activation and the expression of T-bet and Eomes^22^. Thus, IL-2 and IL-15 might be the most important cytokines in γδ T cell-based antitumor immunotherapy. In addition, IL-18 and IL-12 are involved in IFNγ production in γδ T cells^85,86^, while IL-1β and IL-23 promote IL-17 production^42,87^.

## Clinical implications

### Application of γδ T cells as prognostic factors

Bisection of IL-17 and IFNγ levels in γδ T cells can be used for prognostics. IL-17-producing γδ T cells are associated with negative survival outcomes in patients with gallbladder or colon cancer^52,88^. Research has demonstrated that these cells are related not only to survival but also to tumor size, invasion, and metastasis. In contrast, IFNγ-producing γδ T cells tend to be associated with a positive prognosis and prolonged survival^88^. Although one study suggested that γδ T cells are mostly associated with a positive prognosis, this association remains ambiguous because discriminating the effects of γδ T cells from other lymphocytes in bioinformatics data is difficult^9^. Because IL-17 and IFNγ can be produced by other cells, such as CD4/CD8-positive T cells and NK cells, further investigation is required to determine whether the presence of γδ T cells is a better prognostic factor than total cytokine production. Although recent high-throughput methods, such as single-cell RNA sequencing (scRNA-seq), cellular indexing of transcriptomes and epitopes by sequencing (CITE-seq), and spatial transcriptomics, are expensive and labor-intensive, using these methods to diagnose patients ultimately provides more insightful information.

### Current strategies for using γδ T cells in the clinic

**Fig. 4:**
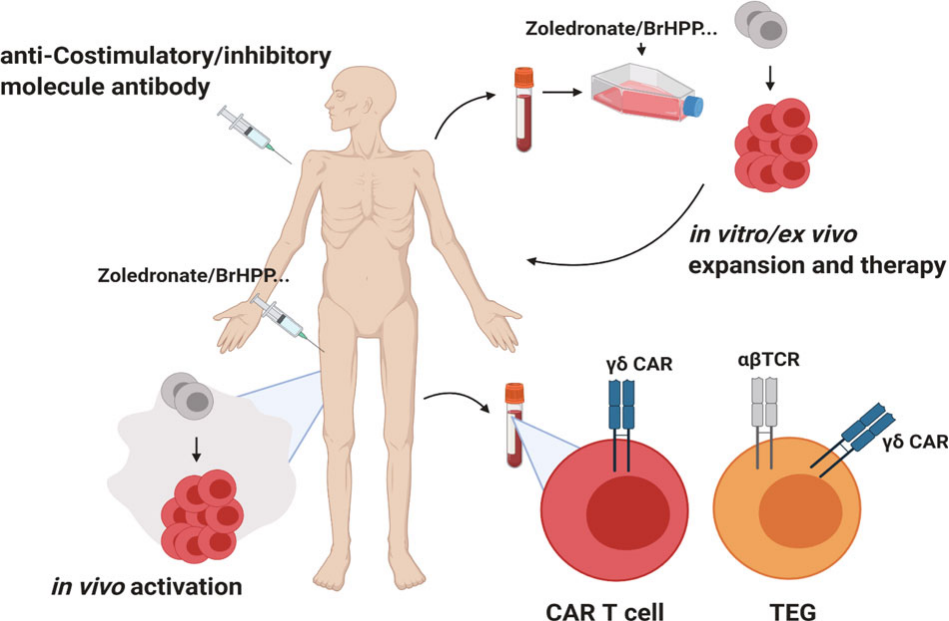
Clinical implication of using γδ T cells. Antitumor therapies using γδ T cells are currently being developed. PBMCs from patients can be expanded under TCR- and cytokine-stimulating conditions. Expanded γδ T cells can kill tumor cells in vitro. Patient-derived cells can be transduced with engineered γδTCR. γδ CAR-T cells have the potential to be used for therapy. γδTCR-transduced αβ T cells (TEGs) are thought to be effective therapeutic agents. Injection of antibodies against costimulatory molecules or anti-inhibitory molecules can be used to reinvigorate γδ T cells. Reagents, such as zoledronate, can be used to activate γδ T cells de novo.

Vγ9Vδ2 T cells are common targets for γδ T cell-based immunotherapies (Fig. 4). These cells are a dominant type of blood-derived γδ T cells, and they are relatively easier to expand in vitro than Vδ1^+^ T cells^7^. One traditional strategy for using γδ T cells is adoptive cell therapy. Stimulation of γδ T cells using aminobisphosphonates or synthetic phosphoantigen analogs can induce Vγ9Vδ2 T-cell expansion in vitro and ex vivo. Aminobisphosphonates, such as pamidronate and zoledronate, act as ligands for γδTCR to upregulate the mevalonate pathway and induce the production of pyrophosphate intermediates in cancer and myeloid cells. Synthetic phosphoantigen analogs, including bromohydrin pyrophosphate (BrHPP) and 2-methyl-3-butenyl-1-pyrophosphate (2M3B1PP), also directly act as ligands for γδTCR^89^. Adoptive transfer of expanded Vγ9Vδ2 T cells was shown to be safe; however, the results from these studies have been disappointing. Several reasons for these failures have been suggested. First, the Vγ9Vδ2 TCR repertoire is too polyclonal to recognize tumors. Because recent studies have identified a novel γδTCR ligand, selecting case-matched ligands for expansion is needed. For example, brain tumor cells express Annexin-A2, which can be targeted by γδ T cells^90^. However, additional studies are needed to examine whether Annexin A2-stimulated γδ T cells are more efficient than traditional-drug-stimulated γδ T cells. Second, the TME can induce γδ T cells to dysfunctional and exhaustion. Oxygen tension and metabolic state can independently affect γδ T-cell function. GBM-infiltrating γδ T cells are prone to becoming more apoptotic and dysfunctional as tumors progress^28^. Thus, deletion of endogenous γδ T cells did not affect the overall survival of GBM-bearing mice. Furthermore, although the adoptive transfer of γδ T cells ex vivo expanded extended the survival of human GBM-bearing immunodeficient mice, treatment with expanded murine γδ T cells was not beneficial for syngeneic GBM-bearing immunocompetent B6 mice^28^. This result suggests that γδ T cells are dramatically immunosuppressed in the TME. One study showed that hypoxia upregulates the cytotoxicity of γδ T cells in vitro. However, the survival of γδ T cells is downregulated by hypoxia^91^. Another study showed that hypoxia inhibits tumor-derived exosome-mediated γδ T-cell activation^26^. However, physiological normoxia (6% oxygen) is hypoxic compared to the 20–21% oxygen levels of the in vitro environment used in this study; therefore, using a more physiologically relevant model system is recommended. Additional studies to understand which γδ T cells are regulated in the TME in vivo are required to develop more effective immunotherapies. Other drugs or therapies can affect γδ T-cell immunity. For example, chemotherapy and radiotherapy not only kill tumor cells but also render γδ T cells fragile^92^. 5-Fluorouracil, doxorubicin, and cisplatin increase tumor cells' sensitivity to γδ T cells^93^. The DNA methylation inhibitor decitabine was reported to upregulate the NKG2D ligand on tumor cells^94^. Thus, to avoid unexpected side effects, the administration of drug combinations should be considered carefully. On the other hand, an adequate combination of conventional drugs may have antitumor effects mediated by γδ T cells. Reinvigorating γδ T cells by targeting coinhibitory/stimulatory molecules might be a good strategy. Anti-CTLA4 antibody treatment increased the frequency of Vδ2^+^ T cells in melanoma patients^95^. However, combination therapy using anti-PD-1 and anti-CTLA4 antibodies produced almost no change in γδ T cell levels^96^. Finding a reasonable target for reinvigorating γδ T cells might be more important before clinical use. Nanobodies or bispecific antibodies have also been used to increase the specificity and activity of γδ T cells^7^.

Vδ1^+^ T cells have been infrequently leveraged as therapeutic targets because validated agonizts for the Vδ1 TCR have yet to be identified. However, a recent study showed that peripheral blood-derived cells can be expanded into Vδ1^+^ T cells in vitro^97^. These cells were termed Delta One T cells. TCR ligation and IL-15 supplementation induce the expression of NKRs such as NKp30 and NKp44. Another study showed that Vδ1^+^ T cells derived in vitro can recognize the melanoma antigens MART1 and gp100. MART1- and gp100-reactive Vδ1^+^ T cells were restricted to HLA-A2. This is the first evidence of MHC restriction in γδ T cells^24^. This knowledge can be used to develop MHC-restricted γδ T-cell therapies and vaccines.

Recently, γδTCR-based chimeric antigen receptor (CAR)-T cells were developed. Although their therapeutic efficacy remains mostly unknown, many people expect γδ CAR T cells to be beneficial in attenuating cytokine release syndrome and neurotoxicity^7^. A recent study showed that γδ CAR T cells are active against leukemia in vitro and in vivo. However, these cells have limited persistence^98^. Because γδ CAR-T cells might be effective alternatives for use against antigen-negative or MHC-low tumor cells, further development and research should be performed. In addition to the cells exhibiting direct cytotoxicity and migration ability, γδ CAR-T cells developed by Capsomidis et al.^99^ showed the cross-presentation ability to T cells. To maximize the advantages of each of these T-cell types, Vγ9Vδ2 TCRs have been transduced into αβ T cells^100^. These cells are termed T cells engineered with defined gamma delta TCRs (TEGs). When γδ TCR genes are transduced into CD4 T cells, CD4 TEG cells exhibit both cytotoxicity and helper activity that can aid in the maturation of DCs. Furthermore, because αβ T cells express low levels of inhibitory KIRs, γδ TCR-mediated cytotoxicity is less inhibited^89^. Furthermore, a recent study has shown that natural TEGs in CNS inflammation environments display enhanced effector functions^33^. If these cells can be isolated or expanded, they can be beneficial for clinical use.

### Future perspectives

Because γδ T cells constitute a minor cell population, their importance in multiple diseases has been neglected. Furthermore, the homologs in animal models and humans do not match. In particular, DETCs do not exist in humans. Because neither information about TCR ligands nor a system for expanding antigen-specific murine γδ T cells in vitro and ex vivo are available, investigating γδ T cells has been relatively challenging. However, recent studies have emphasized the unexpected importance of γδ T cells, specifically in multiple tumor types. Despite the low number of γδ T cells during homeostasis, a large number of γδ T cells are recruited to tumor sites to perform effector functions. In some tumors, γδ T cells exhibit more antitumor activity than conventional T cells. For this reason, we anticipate the development of γδ T cell-based therapies in the future even though clinical results have been disappointing thus far. Several studies have suggested that the roles of γδ T cells are very complicated and context-dependent. Thus, three aspects must be determined to successfully use γδ T cells as therapeutic agents in the clinic. First, we need to identify cognitive murine γδTCR antigens. Although the use of several antigens, including Annexin-A2 and Skint1, has been suggested, expansion of peripheral γδ T cells in vitro has not been realized. As a result, it is difficult to generate ex vivo cultures or perform adoptive transfers using murine γδ T cells in immunocompetent mice. Transfer models of γδ T cells using immunodeficient mice have obvious limitations because the effects of other immune cells on γδ T cells are excluded. Furthermore, tumor antigens that can be recognized by γδ T cells must be identified before γδ T cells can be used for therapy. Second, investigations of γδ T cells in the TME using high-end technology are needed. Recently, scRNA-seq, cytometry by time of flight, and spatial transcriptomics have provided insightful information at the single-cell level. Because γδ T cells are highly heterogeneous, it will be helpful to understand complex γδ T-cell biology. Third, a systemic review of the roles of γδ T cells in a case-by-case manner is needed even though the results from these studies can sometimes be stochastic and inconsistent. Upon completion of the first and second steps, we must summarize and classify the complex γδ T-cell functions. Once we understand more about γδ T cells, they may become game-changing tools in the fight against cancer.
